# Implicitly tuned: The neural effects of social feedback on adolescent learning

**DOI:** 10.1016/j.dcn.2026.101809

**Published:** 2026-09-06

**Authors:** Alexis N. Bosseler, Erica Peterson, Katherine K. Meltzoff, Julia C. Mizrahi, Eric Larson, Neva M. Corrigan, Samu Taulu, Andrew N. Meltzoff, Patricia K. Kuhl

**Affiliations:** a University of Washington, Seattle, WA, United States; b University of California Riverside, Riverside, CA, United States

**Keywords:** Social cues, Adolescents, Implicit Learning, Social feedback, Learning related neural responses, MEG, Auditory statistical learning

## Abstract

Adolescents are particularly tuned to social cues, but how does this sensitivity affect their learning? This study examined whether social feedback conveyed through human faces modulates implicit learning of auditory speech patterns. Magnetoencephalography (MEG) was used to assess neural measures of learning in response to auditory linguistic input as participants engaged in a computer-controlled guessing task and received incidental social feedback in the form of smiling (positive) or frowning (negative) faces. The results showed that smiling faces produced neural response patterns consistent with learning statistical regularities in left-hemisphere regions involved in social and language processing, including the amygdala, anterior auditory cortex, inferior frontal cortex, and insula, whereas no significant learning-related effect was observed in the right hemisphere. In contrast, negative social feedback (frowning faces) did not produce a significant learning-related effect in either hemisphere, indicating no reliable neural effect of learning under negative feedback. Direct comparisons for positive and negative feedback revealed that the neural effect of learning was significantly larger for positive than for negative feedback, with this effect remaining robust after correction for multiple comparisons in the left insula. These results occurred despite the visual cues being incidental and unrelated to the auditory input. Together, the findings suggest that incidental social feedback from human faces influences neural mechanisms of learning during adolescence.

## Highlights

• Smiling faces enhance neural measures of learning in adolescence.
• Frowning faces show no robust neural learning effect in adolescence.
• Incidental facial cues modulate implicit learning in adolescents.

## Graphical Abstract

**Figure:**
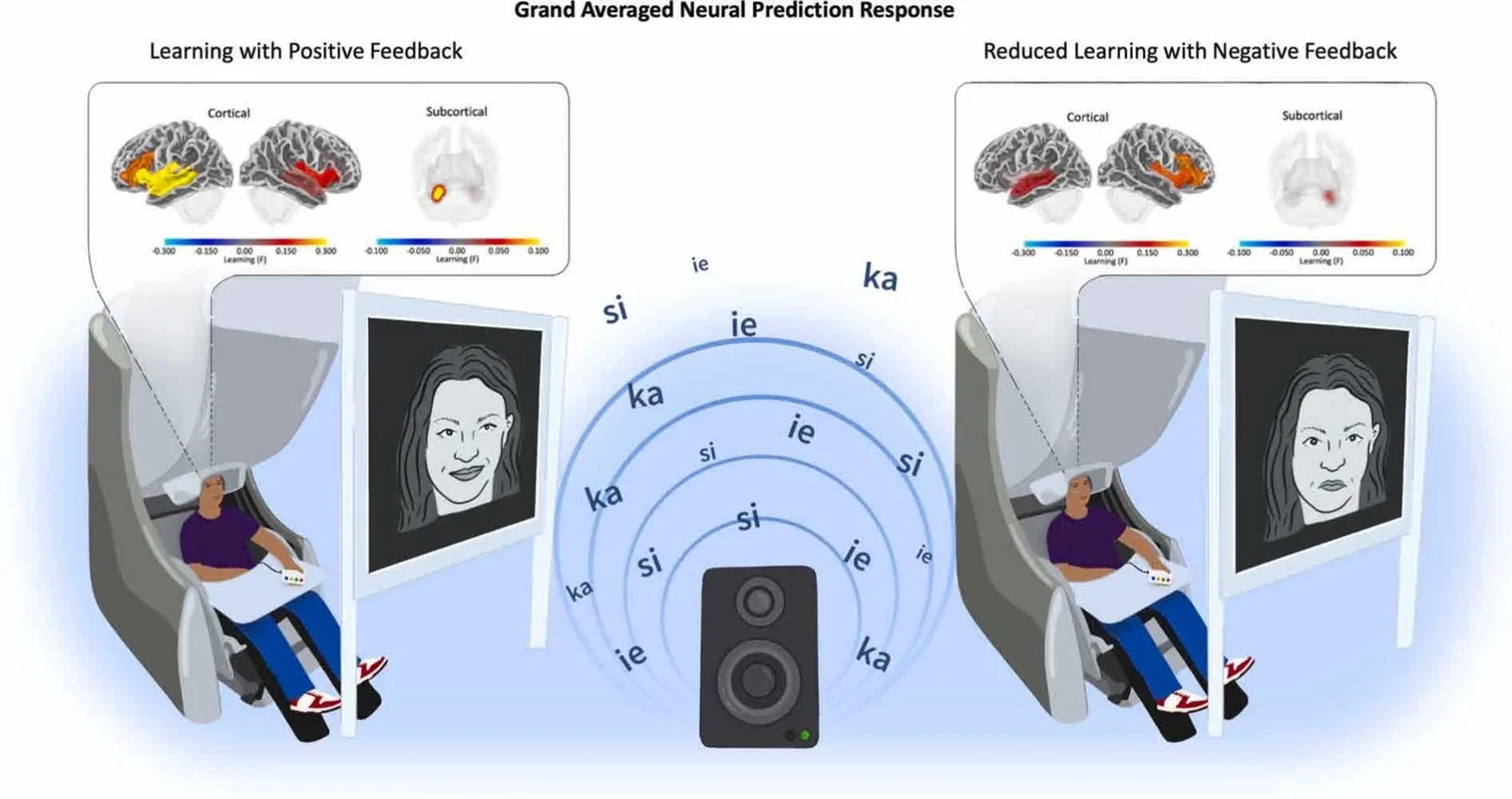

## 1 Introduction

Human learning is not isolated from the social context in which it occurs—it is “calibrated” by it (e.g., Atzil et al., 2018; Livingston and Happé, 2021; Rudolph et al., 2021). From infancy through adolescence, individuals continually adjust their attention, behavior, and cognition in response to subtle social signals (Blakemore, 2008, Blakemore and Mills, 2014, Mills et al., 2021, Sherman et al., 2016, Sherman et al., 2018, Welborn et al., 2016). This process of social calibration—the alignment of internal cognitive processes with external social feedback—involves gauging the surrounding emotional context and adjusting one’s responses accordingly. In developmental neuroscience, growing evidence suggests that implicit learning processes, considered automatic and not under conscious control, are sensitive to ambient social signals (Crone and Dahl, 2012, Mills et al., 2021, Tricoche et al., 2020, Van Hoorn et al., 2018).

Implicit learning is a domain-general, evolutionarily conserved process that enables individuals to extract patterns and regularities from the environment without conscious awareness (e.g., Frost et al., 2015). A well-studied example is statistical learning, which involves detecting statistical patterns—such as transitional probabilities—within continuous streams of input (Saffran et al., 1996). This capacity has been documented across sensory modalities (Conway and Christiansen, 2005, Kirkham et al., 2002) and species (Abe and Watanabe, 2011, Hauser et al., 2001, Lu and Vicario, 2014), underscoring its ubiquity and adaptive significance (Armstrong et al., 2017). Newborn infants are sensitive to statistical structure in both speech (Bosseler et al., 2016, Kudo et al., 2011, Teinonen et al., 2009) and visual input (Bulf et al., 2011). These implicit learning abilities play a foundational role in early cognitive development, allowing infants to extract structure before the emergence of more explicit learning strategies (Fiser and Aslin, 2002). Although especially important in infancy, statistical learning remains active throughout the lifespan (Turk-Browne et al., 2005), helping individuals to adapt to regularities in dynamic environments (Armstrong et al., 2017). Importantly, the efficiency and outcomes of this basic capacity for statistical learning are not fixed; they are shaped by developmental stage and contextual factors, including the social conditions under which learning occurs (Kuhl et al., 2003, Quadrelli et al., 2020).

Adolescence is a period in development characterized by social sensitivity and heightened neuroplasticity (Blakemore and Mills, 2014, Welborn et al., 2016). Adolescents shift toward peer-focused relationships, spending more time in collaborative settings (Brechwald and Prinstein, 2011, Richards et al., 1998, Steinberg and Morris, 2001). They become more attuned to peer feedback and emotionally expressive cues (Cascio et al., 2015, Falk et al., 2014, Rosen et al., 2018). This heightened sensitivity coincides with changes in a network of interconnected brain regions that includes the amygdala (Guyer et al., 2008, Nelson et al., 2014), insula (Tan et al., 2014), and inferior frontal cortex (IFC) (Guyer et al., 2012), which becomes increasingly attuned to peer evaluation and more responsive to social rewards (Blakemore, 2012, Braams and Crone, 2017, Somerville, 2013) sometimes referred to as the ‘social brain’ (Blakemore, 2008).

During adolescence, concerns and worries about social interactions, rejection, and social status become more salient (Haller et al., 2014, Rosen et al., 2018). Subtle social cues, such as a raised eyebrow, a smile, or a frown, take on heightened importance, shaping not only behavior but also how adolescents interpret information in their environment (Casey et al., 2008, Somerville et al., 2010). These findings prompt an important but understudied question: How do different types of social cues affect learning in adolescence, particularly for implicit mechanisms such as statistical learning?

Previous EEG and fMRI research has demonstrated that stimulus salience and emotional context significantly influence learning (Jones et al., 2014, Somerville, 2013). Studies indicate that both positive and negative emotional expressions, particularly those conveyed through facial expressions, can influence implicit learning. For example, adult participants exposed to visual sequences of happy (or sad) faces tend to learn these sequences more effectively than sequences involving neutral faces or simple shapes (Plate et al., 2022). What we do not know is whether emotionally salient faces—when present in the environment as incidental feedback to the material being learned—impact the efficiency of implicit statistical learning, particularly during adolescence.

### 1.1 The current study: incidental social cues, implicit learning, and adolescents

In the present study, we focused on an *a priori* set of regions implicated in auditory statistical learning and social-salience processing. The auditory cortex and surrounding superior temporal regions have been consistently linked to the detection of statistical regularities in continuous speech streams (Cunillera et al., 2009, Frost et al., 2015, Karuza et al., 2013, McNealy et al., 2006, McNealy et al., 2010). In contrast, the amygdala, insula, and inferior frontal cortex (IFC) are core nodes of the adolescent “social brain” that support the processing of affectively salient facial cues, peer evaluation, and social reward (Bickart et al., 2014, Blakemore, 2008, Blakemore, 2012, Guyer et al., 2008, Guyer et al., 2012, Menon and Uddin, 2010, Nelson et al., 2014, Pessoa and Adolphs, 2010, Tan et al., 2014, Uddin et al., 2017). Based on this prior work, we selected the auditory association cortex (AAC) as a primary region of interest (ROI) for tracking auditory statistical learning and the amygdala, insula, and IFC as candidate regions through which incidental social feedback might modulate this learning signal in adolescence.

We examined whether incidental social cues—smiling or frowning faces—modulate adolescents’ statistical learning during an implicit auditory pattern-tracking task. We focused on mid-to-late adolescence because this period represents a developmental stage when youth are particularly attuned to social feedback and highly sensitive to peer evaluation (Crone and Dahl, 2012, Silk et al., 2012, Somerville, 2013, Stroud et al., 2009). We hypothesized that social feedback enhances statistical learning by engaging social- and salience-related regions such as the amygdala, IFC, and insula, particularly when the social information is positive (smiling faces) rather than negative (frowning faces).

To test our hypotheses, we used magnetoencephalography (MEG) combined with a high-resolution T1-weighted structural MRI for co-registration to measure neural responses to auditory statistical input. Participants were exposed to an artificial language stream while receiving incidental social feedback in the form of smiling or frowning faces. This passive auditory stream consisted of tri-syllable pseudowords following a classic statistical learning paradigm (Saffran et al., 1996). This paradigm leverages the contrast between high transitional probabilities within pseudowords and low probabilities between pseudowords, such that the first syllable of each pseudoword reliably signals word onset.

A standard neural marker used to index statistical learning is an enhanced brain response to word-initial syllables (hereafter, ‘s1’) relative to the average of word-medial and final syllables (hereafter, ‘s2/3’) within the 400–600 ms window following the onset of each new pseudoword (Abla et al., 2008, Bosseler et al., 2016, Cunillera et al., 2006). MEG is especially suitable for this research, providing millisecond-level temporal resolution and good spatial localization (Teinonen and Huotilainen, 2012). This allows us to track rapid neural responses to statistical structure and social feedback in real time, and to localize these effects within predefined brain ROIs.

## 2 Methods

### 2.1 Participants

Forty-three participants (20 female) aged 15 and 17 years, were included in the analysis; this sample was drawn from a larger study on adolescent brain imaging. Participants were recruited through the University of Washington Communications Studies Participant Pool. The original study recruited 167 adolescents (81 female) across five age groups, with initial ages of 9, 11, 13, 15, and 17 years. In this larger study, participants had to be right-handed (Edinburgh Handedness Scale score of 80% or higher), native English speakers, and monolingual. Exclusion criteria included a history of neurological, psychiatric, learning, or speech and hearing disorders, as well as non-removable body metal (preventing an MRI). These were assessed via self-report using a standard screening questionnaire at recruitment and verified with a follow-up questionnaire after enrollment. Orthodontia-related signal degradation in MEG and MRI data also served as an exclusion criterion. Finally, four participants began the experiment but were excluded for the following reasons: excessive movement during imaging (*n* = 3) or difficulties with task instructions (*n* = 1).

### 2.2 MRI data acquisition

Magnetic Resonance Imaging (MRI) data were acquired on a 3.0 T Philips Ingenia MRI system using a 32-channel head coil. A Pearltec Crania (Pearltec AG, Schlieren/Zurich Switzerland) head fixation system was used to minimize head motion. High-resolution T1-weighted images of the head were acquired using a multi-echo MPRAGE sequence with FOV = 240 × 240 x 200, acquisition voxel size 1.0 × 1.0 × 1.0 mm^3^, reconstructed voxel size.5 x.5 x.5 mm^3^, TR/TI/TE1/TE2 = 13.6/1, 100/3.7/9.8 ms, shot interval 2200 ms, and flip angle (FA) = 12°.

MRIs were segmented using FreeSurfer version 7 (Fischl, 2012) to generate volumetric and cortical parcellations along with surfaces for the outer skin, skull, pial, gray, and white matter. Individual single-layer boundary element models were constructed from the inner skull surface (Mosher et al., 1999). Because our regions of interest included both cortical and subcortical areas, we created mixed source space for forward modeling, including cortical regions (AAC, insula, IFC) and a subcortical region (amygdala). Individualized surface source spaces were created by evenly distributing 4098 candidate dipole locations per hemisphere along the gray-white matter boundary. In addition, volumetric source spaces, with dipoles distributed in a 4 mm grid constrained beneath the cortical surface, were derived for each participant, resulting in a mixed source space with both surface and volumetric candidate dipoles. Dipoles were loosely constrained to be normal to the cortical surface. Manual co-registration of MRIs to the MEG coordinate space was performed using MNE-Python (Gramfort et al., 2013).

### 2.3 MEG data acquisition

All recordings were collected inside a magnetically shielded room at the Institute for Learning and Brain Science (I-LABS) at the University of Washington. Data were recorded with an Elekta Neuromag 306-channel whole-head magnetoencephalography system (VectorView™, Elekta Neuromag Oy, Helsinki, Finland). Data were sampled at 1000 Hz with a high-pass filter at.03 Hz. Limb-lead ECG, as well as vertical and horizontal EOG, were collected throughout the recording. Five continuous head position indicator (cHPI) coils were affixed around each subject’s head on the scalp near the hairline, and signal from the coils was used to continuously track each subject’s head position throughout the recording. A Polhemus FastTrak™ 3D position monitoring system (Polhemus, Colchester, VT) was used to digitize coil positions, locations of fiducial points including the nasion as well as left and right auricular points, and additional points (at least 200) covering the surface of the scalp.

Participants were comfortably seated in an upright position throughout the recording. An MEG technician monitored all recordings for data noise and artifacts, manually marking noisy or saturated channels detected via visual inspection, ensuring participant compliance, and monitoring SNR of the cHPI coils.

### 2.4 Stimuli and experimental procedure

**Fig. 1:**
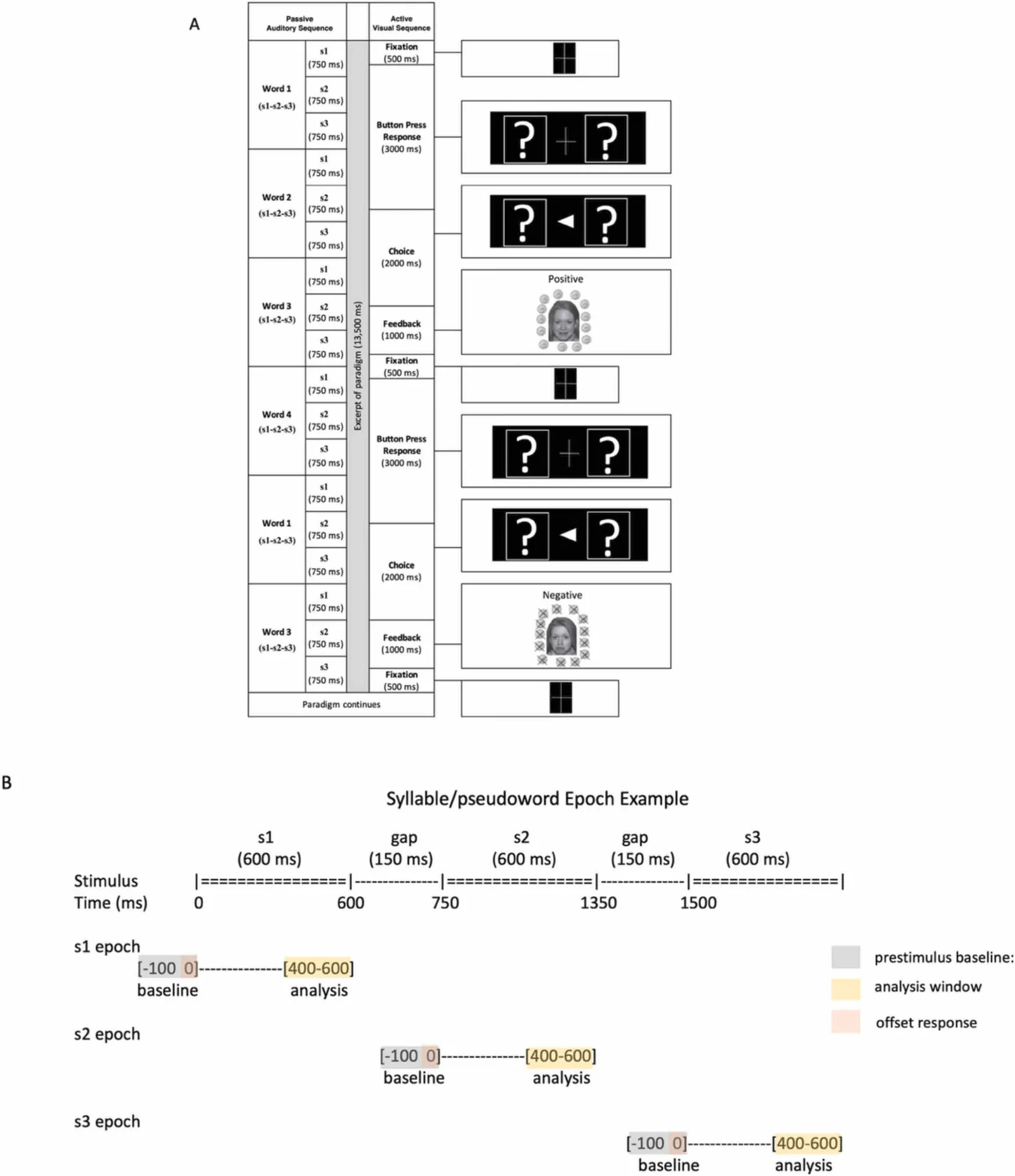
Experimental task design and stimulus timing. Participants passively listened to a continuous auditory speech stream consisting of tri-syllabic pseudowords while simultaneously performing a visual two-alternative forced-choice task. (**A**) **Task Design–**Left column (passive auditory stream): The speech stream consisted of pseudowords with three-syllable positions. Each syllable had a duration of 600 ms with a 150 ms period of silence between syllables. Neural responses were epoched relative to the onset of each auditory syllable (0 ms) with a window of −100–750 ms. Responses were later categorized according to the valence (positive vs. negative) of the most recently presented feedback in the visual task. **Task Design–**Right column (active visual stream): Each trial began with a fixation cross (500 ms), followed by a choice screen displaying question marks (3 s). Participants indicated their choice (left or right) using a MEG-compatible button response pad (Cedrus RB-series response pad) with their left thumb. After the response, a confirmation arrowhead appeared (1 s), followed by social feedback presented as a smiling or frowning face (1.5 s). The auditory speech stream continued uninterrupted throughout the task and was not temporally synchronized with the visual events. Visual stimuli were projected onto a screen positioned in front of the participant inside the magnetically shielded room. **(B) Timeline of the Statistical Learning Stream and Baseline/Epoch Overlap.** Syllables were presented in a rapid, continuous serial stream with an acoustic duration of 600 ms (indicated in blue), separated by a fixed 150 ms silent inter-syllable interval. The target cognitive analysis window is 400–600 ms, indicated in yellow. As illustrated by the vertical alignment (shaded orange), the peristimulus baseline window (-100–0 ms) for any given syllable (n) maps directly onto the 600–750 ms post-stimulus offset/silent window of the preceding syllable (n-1). Control analyses performed on the raw prestimulus interval revealed no reliable condition differences prior to baseline correction.

Participants passively listened to a speech stream while completing a two-alternative forced-choice task that elicited, under preprogrammed computer control, social feedback via images of either happy or sad faces. A schematic of the task design and stimulus timeline is shown in Fig. 1.

#### 2.4.1 Active visual sequence

The active visual sequence task is described in detail in Stavropoulos and Carver (2014). Briefly, the visual sequence proceeded as follows. At the beginning of each trial, a fixation cross appeared on screen for 500 ms. After the fixation cross, two boxes were displayed for 3000 ms; each box contained a question mark inside it. Participants were asked to guess which question mark was correct using a MEG-compatible button response pad (Cedrus RB-series response pad) with their left thumb. After participants chose the left or right box, an arrowhead appeared in between the question marks for 2000 ms, indicating their choice (e.g. the arrowhead pointed left or right to indicate the chosen box). After 2000 ms, feedback about whether the participant guessed correctly appeared on screen for 1000 ms, which appeared as a photograph of a face that was either smiling or frowning to indicate positive or negative feedback, respectively, of the participant’s choice.

Visual stimuli were presented using an Epson Home Cinema 3800 projector onto a screen positioned 122 cm from the participant. The main face stimulus was displayed within a square region measuring 52 × 52 cm on the projection screen, subtending approximately 24.1° of visual angle at the viewing distance. The fixation cross, presented centrally prior to stimulus onset, measured 14 × 14 cm (≈6.6°). Feedback displays included coin stimuli with a diameter of 7 cm (≈3.3°). Choice stimuli consisted of square frames measuring 39 × 39 cm (≈18.1°), each containing a centrally positioned question mark symbol approximately 32 cm in height (≈14.9°). All stimulus dimensions were scaled proportionally from the display layout and were held constant across trials.

To ensure that attention was directed to the visual sequence, participants were given a financial incentive contingent on their performance. Participants were informed that for every “correct” choice, indicated by the image of a smiling face, they would earn a quarter, and that the sum of their earnings would be paid to them by mailed check after the completion of the session. No information was given to the participants about how to choose the “correct” direction. In fact, positive and negative feedback was randomized by computer to ensure ongoing engagement in the task. All participants received $20 for completing the task. At the end of each session, each subject was informed of a putative number of “correct” responses earned during the task, with possible values ranging from 76 to 80, and the participants were told that their check would be rounded up to the next nearest dollar.

#### 2.4.2 Passive auditory sequence

A speech stream consisting of tri-syllabic pseudowords was played from a piezoelectric flat speaker inside the MSR positioned directly in front of the seated participants at a distance of 6 ft. The full details of the auditory stimuli can be found elsewhere (Bosseler et al., 2016).

The speech stream consisted of four pseudowords. The speech stream was statistically manipulated such that syllabic transitions within pseudowords were perfectly predictable, but transitions between pseudowords were equiprobable and unpredictable except that pseudowords were never immediately repeated. For example, pseudoword A was followed by pseudoword B, C, or D an equal number of times, just as pseudoword B was equally followed by A, C, and D, and so on. Thus, transitions following the final syllable of a pseudoword had an equal probability of 33%, while transitions following the initial and medial syllables had probabilities of 100%. Pseudowords were constructed from 36 syllables recorded from a female speaker in an anechoic chamber. Syllables were acoustically matched for duration (600 ms), intensity, loudness, and mean pitch. Each pseudoword consisted of three syllables. Syllables were presented with a 150 ms silent interval between them, yielding a 750 ms onset-to-onset interval between consecutive syllables and a total pseudoword duration of 1800 ms.

### 2.5 Data reduction and analysis

Data were preprocessed and analyzed using MNE-Python (Gramfort et al., 2011). Continuous head position coil frequencies and line noise were filtered with an iterative amplitude fitting method. Extended Signal Space Separation (eSSS) (Helle et al., 2021) with movement compensation was used to clean the data from movement artifacts, external noise fields, and vibration artifacts (eSSS uses the temporal extension of Maxwell filtering with an additional basis field derived from empty room recordings for noise cancellation). Window duration for eSSS was 20 s with a correlation limit of.98 for environmental noise rejection. Channels marked by the MEG technician as bad were reconstructed during the Maxwell filtering step. Using symmetric linear-phase finite impulse response filters with Hamming windows and a filter length of 10 s, clean raw data were band-pass filtered from.03 to 80 Hz. Cardiac and blink artifacts were removed with Signal Space Projection (Nolte and Curio, 1999).

Data were epoched from −100–750 ms after the onset of each auditory syllable. Epochs were time-locked separately to each syllable position (s1, s2, and s3), and the 400–600 ms analysis window was chosen to capture the neural response of interest for each syllable (e.g., McNealy et al., 2006; Takács et al., 2021). Epochs with significant artifacts, defined as having a peak-to-peak amplitude exceeding 3e-10 T/cm for gradiometers or 9e-12 T for magnetometers, were automatically detected and dropped. Epochs were baselined, and a noise covariance was estimated from −100–0 ms using the “shrunk” estimator (Engemann and Gramfort, 2015). Each participant contributed approximately 136 epochs per condition (s1: *M* = 135.9, *SD* = 3.0; s2: *M* = 136.3, *SD* = 2.9; s3: *M* = 136.3, *SD* = 2.9; range 125–137 in all conditions).

The interstimulus interval (150 ms) was chosen to balance the need to minimize overlap between successive evoked responses with the requirement for temporally contiguous input, which is important for statistical learning paradigms that rely on rapid sequential structure. Given the relatively rapid stimulus presentation (600 ms syllables with a 150 ms interstimulus interval), some overlap between successive evoked responses is unavoidable, and residual carry-over activity from preceding stimuli may contribute to the measured signal. To evaluate whether the observed post-stimulus effects could be explained by systematic differences in prestimulus activity, we analyzed the baseline interval (−100–0 ms relative to syllable onset) using paired *t*-tests. This analysis was performed on the raw (uncorrected) signal prior to baseline correction. Across all tested comparisons, there was no evidence of systematic differences in prestimulus activity between conditions or syllable positions (all *p*’s > .1). Effect sizes were small and not reliably different from zero (all Cohen’s *d* <.15), indicating minimal differences in prestimulus neural activity entering the baseline period. These control analyses were conducted to assess potential confounds related to overlapping responses in rapid stimulus sequences.

In addition, because s2 and s3 were treated as variants of the same syllable type, we averaged the number of epochs across s2 and s3 to form a combined s2/3 syllable type for the analysis. A paired *t*-test comparing the number of valid epochs for s1 with the averaged s2/3 condition revealed no significant difference, *t*(39) = 1.60, *p* = .56, indicating that the conditions were well matched across participants.

A forward model was constructed for each participant from the individualized mixed source space. Source estimates were computed using dynamic Statistical Parametric Mapping (dSPM) as implemented in MNE-Python. dSPM produces noise-normalized source amplitude estimated by whitening the source reconstruction using the noise covariance matrix, resulting in values that reflect signal-to-noise-normalized cortical activity (that can be interpreted as pseudo-*F* statistics) rather than inferential statistics.

Source time courses were extracted from anatomically defined ROIs, including bilateral AAC, insula, and IFC using cortical labels from the Human Connectome Project combined atlas (Glasser et al., 2016), and within the bilateral amygdala using subcortical segmentations derived from FreeSurfer (Fischl, 2012). For each ROI, source time courses were extracted and averaged across all vertices within the ROI to obtain a single representative time course per region. These ROIs (AAC, insula, IFC, and amygdala) were defined *a priori* based on prior work on auditory statistical learning and social-salience processing, and were derived from the HCP combined atlas (cortical ROIs) and FreeSurfer segmentations (amygdala).

For each ROI and hemisphere, we extracted the mean source activity (dSPM value) within a predefined 400–600 ms time window relative to syllable onset. This window was selected *a priori* based on prior work on auditory statistical learning and mid-latency N400-like responses (Abla et al., 2008, Bosseler et al., 2016, Sanders and Neville, 2003a, Sanders and Neville, 2003b, Takács et al., 2021, Teinonen et al., 2009, Teinonen and Huotilainen, 2012), and was applied uniformly across all conditions, ROIs, and hemispheres.

To test whether social feedback modulated the neural measure of learning we examined the effect of syllable position using source-level ROIs with dSPM. Our analysis focused on the time-averaged response from 400 to 600 ms in a set of *a priori* ROIs motivated by prior work on auditory statistical learning and social-salience processing. These included AAC, which is critical for auditory statistical learning (Abla et al., 2008, Bosseler et al., 2016, Cunillera et al., 2009, Frost et al., 2015, Karuza et al., 2013, McNealy et al., 2006, McNealy et al., 2010, Sanders and Neville, 2003a, Sanders and Neville, 2003b, Takács et al., 2021, Teinonen et al., 2009, Teinonen and Huotilainen, 2012), and areas important to processing socially salient facial cues and reward signals—the amygdala (Bickart et al., 2014, Pessoa and Adolphs, 2010), IFC (Aron et al., 2014), and insula (Menon and Uddin, 2010, Uddin et al., 2017)

The dSPM values were analyzed with linear mixed-effects models (LMMs) implemented in JASP (2026), with participant modeled as a random intercept. Participant was modeled as a random intercept to support inference at the population level based on between-subject variability (N = 40), while the large number of epochs per condition served to improve within-subject signal-to-noise ratio. Syllable position (s1 vs. s2/3), feedback type (positive vs. negative), hemisphere (left vs. right), and ROI (amygdala, IFC, AAC, and insula) were entered as fixed effects. Degrees of freedom and *F* statistics for fixed effects in the LMMs were obtained using Satterthwaite’s approximation. When significant interactions were observed, follow-up paired-samples *t*-tests were conducted for ROI-specific comparisons. Model statistics for fixed effects (*β*, *SE*, *F*, and 95% CIs) are reported. Cortical maps (Fig. 3, Fig. 4) illustrate dSPM magnitudes and are not intended to represent statistical contrasts. To address potential developmental effects within our adolescent sample, we also tested whether age (15 versus 17 years) moderated the neural response to learning by including age as an additional fixed factor in the LMMs with syllable position, feedback type, hemisphere, and ROI.

The analyses addressed two related but distinct questions. First, an omnibus LMM tested whether neural responses differed between s1 and s2/3 and whether syllable-position effect varied by feedback condition, hemisphere, or ROI. Significant interactions were decomposed using ROI-specific paired-samples t-tests, which tested the effect of syllable position within each feedback condition and ROI. Second, we directly compared the magnitude of the learning-related response between feedback conditions. For this analysis, the learning effect was defined as the within-subject differences between s1 and s2/3 (s1–s2/3), and this difference score was compared between positive and negative feedback conditions. Multiple-comparison correction was applied to the follow-up comparisons.

All analyses used the predefined ROIs and 400–600 ms time window described above. We report both uncorrected *p*-values and *p*-values corrected for multiple comparisons using the Benjamini–Hochberg FDR procedure (Benjamini and Hochberg, 1995). FDR correction was applied separately within each family of ROI-level comparisons (4 ROIs x 2 hemispheres). Cohen’s *d* is reported as an index of effect size. Because statistical inference was performed on spatially averaged ROI values rather than voxel-wise whole-brain maps, multiple-comparisons correction was applied at the ROI level rather than across the full source space.

Within this framework, a learning-related response was operationalized as a difference in response magnitude between s1 and s2/3 that reflects sensitivity to the statistical regularities in the input. When this s1-s2/3 difference was not statistically significant within a feedback condition, we describe this as no reliable neural effect of learning. No condition-specific peak or sensor selection was performed; instead, statistical analyses were conducted using linear mixed models applied to ROI-level dSPM values derived from source reconstruction.

## 3 Results

**Fig. 2:**
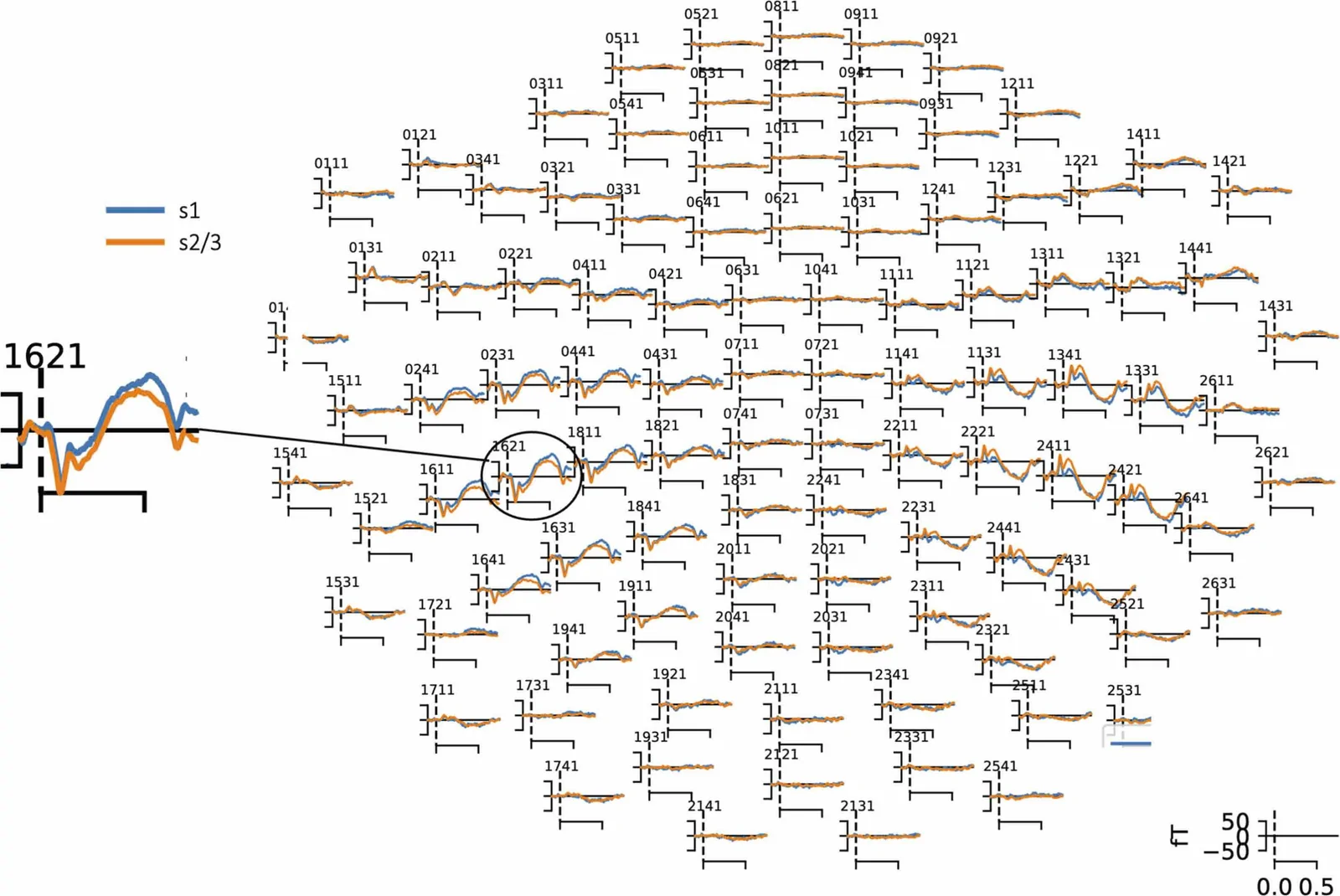
Grand-averaged auditory-evoked waveforms (sensor-level). Waveforms are shown from an example left-hemisphere sensor triplet (one gradiometer pair and one magnetometer). Each waveform is time-locked to the syllable onset (syllable duration = 600 ms; inter-syllable interval = 150 ms; −100–0 ms baseline applied). All reported analyses were performed at the source level.

Fig. 2 shows the grand-averaged waveforms for an example sensor triplet (one gradiometer pair and one magnetometer) from the left-hemisphere temporal region. This figure is provided solely to illustrate data quality and the presence of reliable auditory-evoked activity across participants; all statistical inference was conducted exclusively at the source level.

### 3.1 Main effects and global learning

**Fig. 3:**
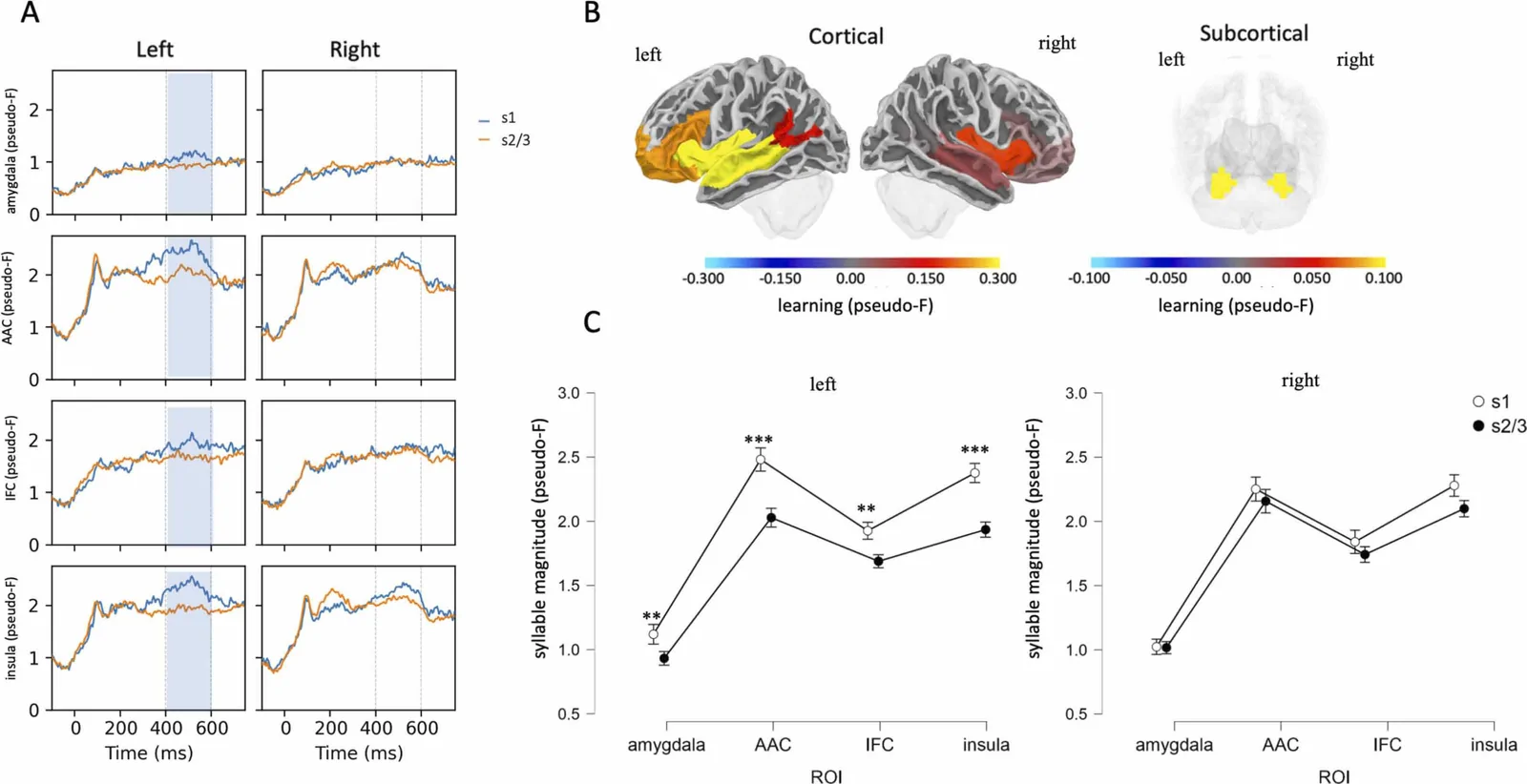
Positive feedback. (A) Grand-averaged source waveforms for the left (left panel) and right (right panel) hemispheres corresponding to the four selected anatomical labels: amygdala, AAC, IFC, and insula. Each plot shows the grand-averaged dynamic Statistical Parametric Mapping (noise-normalized activation pseudo-*F*) values of word-initial syllables (s1, blue line) and the mean of word-medial and -final syllables (s2/3, orange line). (**B**) Grand-averaged source dSPM values showing the difference in activation magnitude between s1 and s2/3, averaged over the 400–600 ms time window. Cortical structures (AAC, IFC, and Insula) are shown in the left panel; the subcortical structure (amygdala) is shown in the right panel. Note: the maps reflect the magnitude of the dSPM response averaged across participants and do not represent statistical contrasts between s1 and s2/3 trials; color scales differ between cortical and subcortical panels (as shown), due to smaller subcortical activations. Critically, the responses across all experimental conditions (s1 vs. s2/3, positive and negative feedback) converge to a similar magnitude during this shared 600–750 ms silence. Although the signal does not return to zero, the similar prestimulus activity across conditions reduces the likelihood that differential carry-over activity accounts for the observed condition effects. (**C**) Mean dSPM values for s1 (open circles) and s2/3 (black circles) are plotted separately for the left hemisphere (left panel) and right hemisphere (right panel). Significant effects of syllable position (s1 versus 2/3) under the positive feedback condition, indicated by *p < .05, **p < .01, ***p < .001, are consistent with neural tracking of statistical regularities in these ROIs.

The LMMs revealed significant main effects of feedback type, *β* = .03, *SE* = .01, *F*(1, 1053) = 5.35, *p* = .02, and syllable position, *β* = .08, *SE* = .02, *F*(1, 39) = 10.25, *p* = .003. Overall, model-predicted response magnitudes were larger for positive than for negative feedback (positive: *M* = 1.81, *SE* =.05, 95% CI [1.71, 1.90]; negative: *M* = 1.75, *SE* =.05, 95% CI [1.66, 1.85]). As shown in Fig. 3, there was also a significant syllable position × feedback type interaction, β = .03, *SE* = .01, *F*(1, 1053) = 6.84, *p* = .009. Within positive-feedback trials, responses were larger for s1 (*M* = 1.91, *SE* =.07, 95% CI [1.78, 2.05]) than for s2/3 (*M* = 1.70, *SE* =.05, 95% CI [1.60, 1.80]), *t*(39) = 3.46, *p* = .001, *p*FDR = .002. In contrast, the effect of syllable position was not significant within negative feedback trials (Δ = 0.09, 95% CI [−0.01, 0.19]), *t*(39) = 1.82, *p* = .080.

**Fig. 4:**
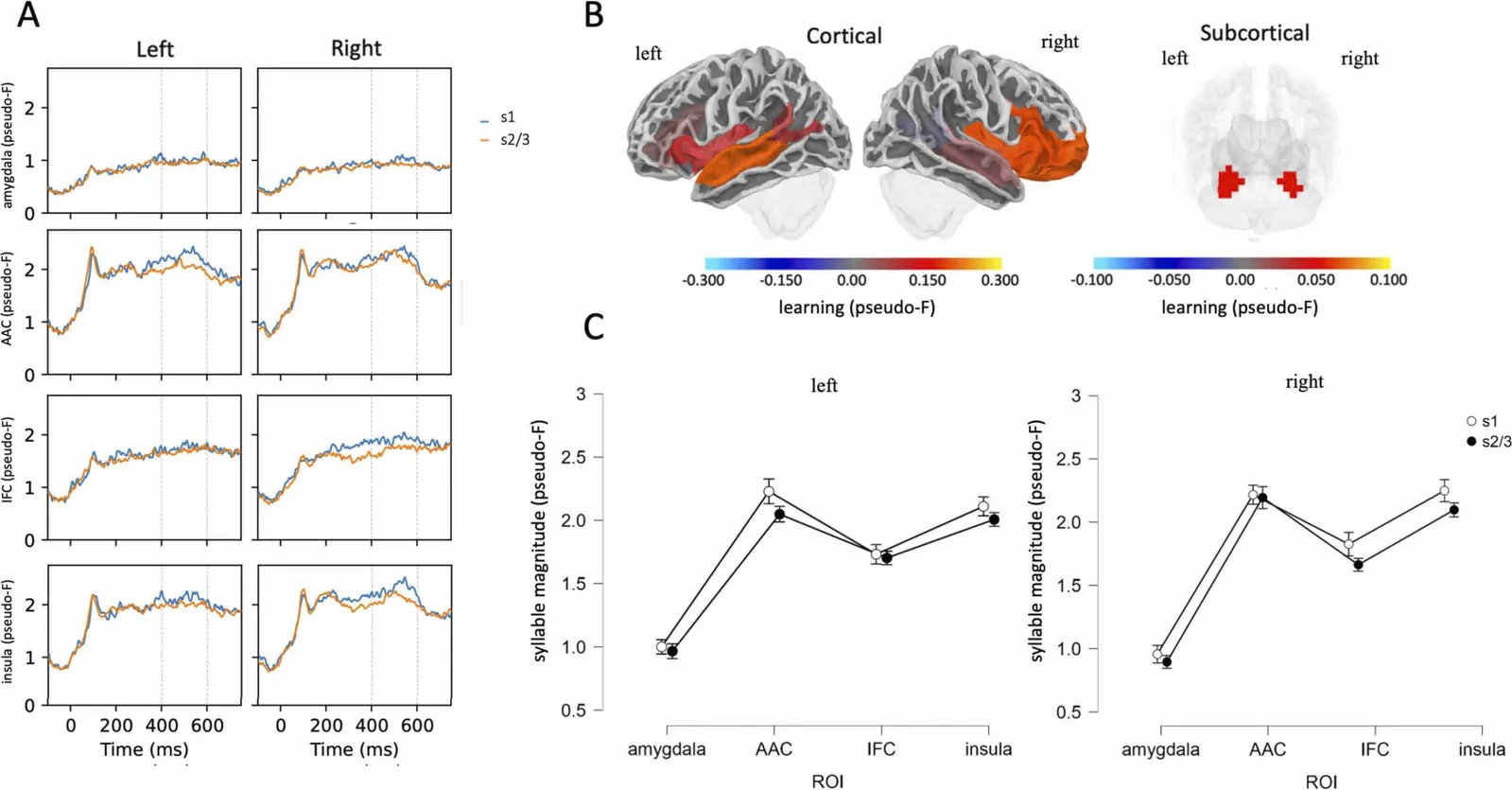
Negative feedback. (A) Grand-averaged source waveforms for the left (left panel) and right (right panel) hemispheres corresponding to the four selected anatomical labels: amygdala, AAC, IFC, and insula. Each plot shows the grand-averaged dynamic Statistical Parametric Mapping (dSPM pseudo-*F* statistic) values of word-initial syllables (s1, blue line) and the mean of word-medial and -final syllables (s2/3, orange line). (**B**) Grand-averaged source dSPM values showing the difference in activation magnitude between s1 and s2/3, averaged over the 400–600 ms time window. Cortical structures (AAC, IFC, and Insula) are shown in the left panel; the subcortical structure (amygdala) is shown in the right panel. Note: the maps reflect the magnitude of the dSPM response averaged across participants and do not represent statistical contrasts between s1 and s2/3 trials; color scales differ between cortical and subcortical panels (as shown) due to smaller subcortical activations. Critically, the responses across all experimental conditions (s1 vs. s2/3, positive and negative feedback) converge to a similar magnitude during this shared 600–750 ms silence. Although the signal does not return to zero, the similar prestimulus activity across conditions reduces the likelihood that differential carry-over activity accounts for the observed condition effects. (**C**) Mean dSPM values for s1 (open circles) and s2/3 (black circles) are plotted separately for the left hemisphere (left panel) and right hemisphere (right panel). The absence of reliable s1 versus s2/3 differences indicates little or no tracking of the statistical regularities in these ROIs.

The LMMs further revealed a significant feedback type × syllable position × hemisphere interaction, *β* = .03, *SE* = .012, *F*(1,1053) = 7.10, *p* = .008. To localize this effect, follow-up ROI-level paired-samples *t*-tests were performed, and FDR correction was applied within each family of ROI-level comparisons (4 ROIs x 2 hemispheres, Table 1). For positive feedback, the effect of syllable position was significant in the l-amygdala, (*t*(39) = 2.90, *p* = .006, *p*FDR = .008, *d* = .46), l-AAC (*t*(39) = 3.91, *p* = 3.57 × 10 −4, *p*FDR = .001, *d* = .62), l-IFC (t(39) = 2.81, *p* = .008, *p*FDR = .0107, *d* = .44), and l-insula (t(39) = 4.35, *p* = 9.42 × 10 −5, *p*FDR = .0004, *d* = .69). In contrast, there were no significant effects of syllable position in the right hemisphere for positive feedback (all *p*’s > .09; Fig. 3), and negative feedback did not produce significant effects of syllable position in any ROI (all *p*’s > .10; Fig. 4). Thus, when presented with negative feedback, these regions showed little to no reliable neural effect of learning, indicating no robust expression of this neural learning marker in the negative-feedback condition.

**Table 1:** Learning-related neural responses by feedback condition. The table reports paired *t*-tests comparing the magnitude of the learning effect (s1 minus s2/3) between positive and negative feedback conditions across ROIs in the left and right hemispheres. Positive values indicate larger learning-related responses following positive feedback. The table provides columns for both uncorrected and FDR-corrected *p*-values (*p*FDR), and Cohen’s *d*.

ROI	Positive Feedback Mean ± *SD*	Negative Feedback Mean ± *SD*	*t*	*p*	*p*FDR	Cohen’s *d*
Left Hemisphere						
amygdala	.20 ±.41	.04 ±.40	2.031	.04	.13	.32
AAC	.45 ±.73	.18 ±.67	2.10	.04	.13	.33
IFC	.24 ±.53	.03 ±.56	1.77	.09	—	.28
insula	.44 ±.64	.10 ±.60	3.09	.004	.03	.69
Right Hemisphere						
amygdala	.01 ±.50	.06 ±.53	-.42	.70	—	-.07
AAC	.09 ±.78	.02 ±.59	.72	.51	—	.11
IFC	.10 +.65	.16 +.60	-.50	.62	—	-.08
insula	.18 +.66	.15 +.55	.67	.80	—	.04

### 3.2 Age effects

Including age (15 vs. 17 years) as a factor revealed a significant age × feedback interaction (*F*(1,1064) = 6.38, *p* = .012). Estimated marginal means showed that 15-year-olds responded similarly to positive and negative feedback (positive *M* = 1.82, *SE* =.06; negative: *M* = 1.81, *SE* =.06), whereas 17-year-olds showed larger responses to positive than negative feedback (positive: *M* = 1.78; *SE* =.08; negative: *M* = 1.66, *SE* =.08). However, age did not interact with syllable position (all *p*s > .15), indicating that the pattern of syllable-position effects on neural response––a marker of learning––was comparable in 15-and 17- year-olds. All other interactions involving age were not significant (all *p*s > .4).

### 3.3 ROI-level learning effects for positive vs. negative feedback

**Fig. 5:**
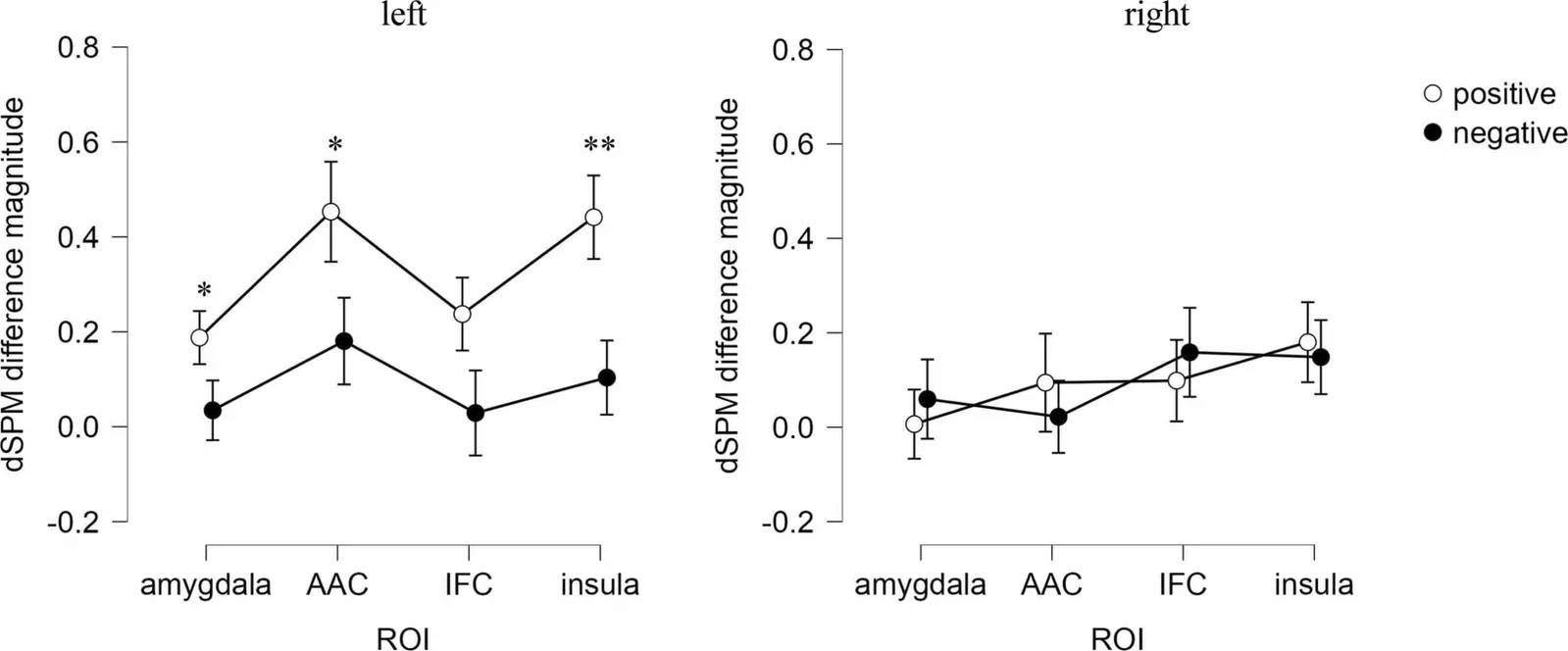
Neural response differences (s1-s2/3) across ROIs and hemispheres for positive and negative feedback. Mean difference in the grand-averaged dynamic Statistical Parametric Mapping (dSPM pseudo-*F* statistic) magnitude between the first syllable (s1) and s2/3 and across four ROIs (amygdala, AAC, IFC, and insula) and left and right hemispheres. Error bars represent ±1 standard error of the mean across participants. Positive feedback trials (white circles) show robust learning-related increases in the s1-s2/3 difference in the left hemisphere auditory regions, whereas negative feedback trials (black circles) show no robust evidence of this neural marker of learning. Statistical effects from the repeated-measures are reported. *p < .05, ** *p* < .01, uncorrected.

To directly test whether the magnitude of the learning-related response differed between feedback conditions, we compared the s1 − s2/3 difference between positive- and negative- feedback trials within each ROI (Fig. 5). In the l-insula, the LMM-estimated learning effect was markedly larger for positive feedback (Δ s1 − s2/3 ≈.42; 2.34 − 1.92) than for negative feedback (Δ ≈.09; 2.10 − 2.01). Consistent with this, paired *t*-tests on the s1 − s2/3 difference showed that the learning-related effect was significantly larger for positive than negative feedback in the l-insula (*t*(39) = 3.09, *p* = .004, *p*FDR = .032, *d* = .69). Importantly, this reflected a clear positive-feedback learning effect and an effect near zero for negative feedback, consistent with the observation that learning-related responses to negative feedback did not reliably emerge across participants.

A similar, though weaker, pattern was evident in the l-amygdala and l-AAC. In the l-amygdala, the model-based learning effect was larger for positive feedback (Δ ≈.15; 1.07 −.93) than for negative feedback (Δ ≈.06; 1.00 −.94), and in the l-AAC, the positive-feedback learning effect was also larger (Δ ≈.43; 2.42 − 1.99) than the negative-feedback effect (Δ ≈.16; 2.19 − 2.03). Subject-level paired *t*-tests showed that these positive-versus-negative differences were significant at the uncorrected level (l-amygdala: *t*(39) = 2.03, *p* = .049, *p*FDR = .13, *d* = .33; l-AAC: *t*(39) = 2.10, *p* = .04, *p*FDR = .13, *d* = .34), although they did not survive FDR correction. Notably, in all ROIs, only the positive feedback condition showed evidence of a learning-related response, whereas the negative feedback condition again showed numerically small, non-significant effects.

No significant difference between positive and negative feedback was observed in the l-IFC at either the model-based or subject-level index (Δ s1 − s2/3 positive ≈.19 vs negative ≈.05; *t*-test *p* = .09, *p*FDR =.28). Consistent with the absence of syllable-position effects in the right hemisphere, no significant differences between positive and negative feedback were observed (all *p*s > .50). These results indicate that, under positive feedback, several left-hemisphere ROIs consistently differentiate between s1 and s2/3, supporting neural tracking of statistical regularities, whereas under negative feedback, these regions show minimal or no such pattern, indicating no robust expression of this neural marker of learning. The l-insula showed the most robust positive-versus-negative difference, remaining statistically significant after correction for multiple comparisons.

## 4 Discussion

We examined the impact of incidental social feedback on learning during adolescence, a period marked by heightened social awareness, using MEG brain imaging. In a classic statistical learning task with auditory speech stimuli, where learning is implicit and expected to be largely automatic**,** we found that positive social feedback in the form of a smiling face produced a neural pattern consistent with learning in brain regions associated with social information processing. These regions included the amygdala, AAC, IFC, and the insula—core nodes of the adolescent ‘social brain’ network. In other words, an incidentally presented smiling face resulted in a neural signature of learning in the same brain regions that process social stimuli. In contrast, negative social feedback in the form of a frowning face showed little to no reliable neural effect of learning in the same brain regions. These effects of positive feedback on learning were observed exclusively in the left hemisphere. This left-lateralized pattern is consistent with our *a priori* focus on regions supporting both language-related auditory statistical learning (AAC) and socially salient processing (amygdala, insula, IFC) in the left hemisphere during adolescence. Together, these findings suggest that social cues influence the detection of statistical regularities in real time, even in tasks in which the social information is incidental.

Examining our neural-based measure of statistical tracking, the present findings show that several left-hemisphere ROIs exhibit reliable s1 versus s2/3 differences under positive feedback, but not under negative feedback. The cortical maps and ROI analyses both indicate that, under negative feedback, responses in the targeted brain regions remain near baseline across syllable positions, providing little evidence of sensitivity to the underlying statistical regularities. These findings indicate that positive social feedback is accompanied by robust cortical tracking of statistical regularities, particularly in the l-insula, an area linked to emotion processing, the integration of sensory and emotional information, and the regulation of attention. The involvement of the l-IFC, functionally aligned with Broca’s area, has been argued to play a role in learning from others, including listening to speech that conveys intent (Hari and Kujala, 2009, Pulvermüller and Fadiga, 2010). The current findings expand on this idea by demonstrating that the l-IFC is more strongly recruited when tracking the statistical regularities in the speech stream while receiving positive, as opposed to negative, social feedback from a human face. Together, the engagement of the l-amygdala, insula and IFC during positive feedback is consistent with the adolescent ‘social brain’ network implicated in processing affectively salient facial cues and social reward, while AAC supports the concurrent tracking of auditory statistical regularities.

The pattern of findings in adolescents aligns with research suggesting that social information can influence learning during brain development. Infant studies show that learning during this time is improved when input is presented with social or emotionally meaningful cues. Young infants better track statistical patterns when exposed to exaggerated prosodic contours typical of infant-directed speech (Bosseler et al., 2016, Thiessen and Saffran, 2003). American infants exposed to Mandarin Chinese in live social interaction demonstrate strong speech-sound learning, whereas those exposed to the same auditory stream via a 2-dimensional, video screen show no learning (Conboy et al., 2015, Conboy and Kuhl, 2011, Kuhl et al., 2003; see also speech vs. nonspeech patterns: Kuhl et al., 1991). Infant neuroimaging research shows that live, face-to-face social interaction activates brain areas involved in attention and speech processing, and that individual differences among infants in the level of neural activation to such social signals predict their course of language development out to 3 years of age (Bosseler et al., 2024). The current findings suggest that, in adolescence, social feedback can modulate neural responses to linguistic regularities, consistent with theories proposing social influences on language learning and processing across the lifespan (Bruner, 1983, Kuhl, 2007, Vygotsky, 1962).

Given the relatively narrow age range of the sample (15–17 years), the present study was not designed to test developmental changes during adolescence. Rather, it aimed to characterize how social feedback influences neural responses to auditory statistical structure during mid-adolescence. The current data provide evidence that feedback from human faces can modulate adolescents’ neural tracking of statistical regularities in an auditory speech stream, suggesting that socially salient feedback can influence ongoing sensory learning processes.

Over the past decade, adolescents’ social environments have changed substantially (e.g., Crone and Konijn, 2018; Maza et al., 2023), with frequent exposure to rapid and repeated streams of social feedback in online environments. Such feedback often comes in the form of symbolic cues—such as emoji, reaction icons, or facial images—that signal approval, disapproval, or emotional tone. Because adolescents regularly encounter and use these symbols in everyday communication, it is important to reconsider how repeated exposure to emotionally salient feedback may interact with ongoing learning processes. Future research could examine how exposure to emotionally charged feedback—such as facial expressions or emoji-based reactions in online environments—affects implicit learning in real time and influences longer-term outcomes related to learning, motivation, memory, and well-being.

The results suggest that negative feedback can impair learning, at least in the task we used, possibly by shifting attention toward emotionally charged but non-task-relevant stimuli. This aligns with a broader body of research showing that, from infancy, humans are particularly sensitive to negative social cues, which can draw attention and affect memory (Carver and Vaccaro, 2007). The salience of negative feedback, especially angry faces, is thought to stem from evolved threat responses, which heighten awareness of possible dangers and improve our ability to plan for avoiding them (Mallan et al., 2013, Öhman et al., 2001, Öhman and Mineka, 2001, Robertson et al., 2023). On the surface, our results differ from those reported by Plate et al. (2022) in adults, where both positive and negative facial sequences enhanced learning. A key distinction is that in our paradigm, the emotional faces were incidental to the auditory statistical learning task—they provided feedback but were not the target of learning. Plate and colleagues’ adults were learning the statistical structure of the visual face sequences themselves, making the emotional content directly meaningful and task-relevant. As a result, negative faces in their study were remembered and influenced learning, whereas in our adolescent sample, the incidental negative faces elicited little to no reliable modulation of auditory statistical learning, likely because they were not directly predictive of auditory patterns. This difference highlights that the effect of emotional context on statistical learning may depend on whether the emotional stimuli are integral to the learning task or merely incidental. It also suggests that adolescents’ learning may be particularly sensitive to positive social feedback when it occurs incidentally, consistent with evidence that positive social signals are especially salient during mid-adolescence (Somerville, 2013, Jones et al., 2014).

Despite its effectiveness in capturing rapid neural dynamics across temporal and spatial domains, MEG remains underused in developmental research. Although solutions for issues such as head movement, motion artifacts, and compliance have been developed for standard SQUID-based MEG systems (Hari et al., 2018, Helle et al., 2021, Taulu et al., 2004, Taulu and Hari, 2009, Taulu and Simola, 2006), and have been successfully applied to developmental MEG data (Clarke et al., 2022, Clarke et al., 2022), concerns about movement-related artifacts still act as a limitation in collecting developmental MEG data (e.g., Coleman et al., 2026; Rhodes et al., 2024), restricting wider use. Over the past two decades, advances in data acquisition, preprocessing, and experimental design have been successfully applied to MEG developmental studies (e.g., Bosseler et al., 2013, Bosseler et al., 2021, Bosseler et al., 2024; Cheour et al., 2004; Imada et al., 2006; Khan et al., 2018; Kitzbichler et al., 2015; Kuhl et al., 2014; McCusker et al., 2021; Meltzoff et al., 2018; Ott et al., 2021; Sambeth et al., 2006). These advances eliminate post-hoc trial exclusions, which can introduce bias, reduce statistical power, and undermine replicability. Leveraging MEG brain imaging during adolescence, combined with appropriate pre- and post-processing methods, therefore, offers a unique opportunity to address salient questions about how sensory and cognitive computations, and their modulation by social context, unfold in real time during critical developmental windows.

Several limitations should be noted when interpreting the present findings. First, although the sample size (*N* = 40) is comparable to many MEG studies and each participant contributed a large number of trials per condition, the overall sample remains modest, and future work with larger samples will be important to confirm the robustness and generalizability of these effects. Second, the primary analyses focused on a small set of *a priori* hypothesized ROIs motivated by previous work on auditory statistical learning and social-salience processing. These ROIs (AAC, amygdala, insula, IFC) were selected to index the interaction between auditory statistical learning and the adolescent social brain. Because these regions and the analysis window were defined independently of the contrasts tested, we treated these analyses as planned tests but nonetheless applied multiple-comparisons correction across ROIs and hemispheres. While this hypothesis-driven approach is common in ROI-based neuroimaging studies (e.g., Kriegeskorte et al., 2009; Rosen et al., 2018; Saxe et al., 2006), replication will be important to confirm the observed effects.

Third, given the relatively rapid stimulus presentation (600 ms syllables with a 150 ms inter-stimulus interval), some overlap between successive evoked responses is unavoidable, and residual carry-over activity from preceding stimuli may contribute to the measured signal. Given the short interstimulus interval, the −100–0-ms baseline period may contain residual activity associated with the preceding syllable. The experimental conditions were randomized and presented with equal frequency, reducing the likelihood of systematic associations between the current condition and the preceding stimulus. Consistent with this, our analysis of the raw pre-stimulus interval found no evidence of systematic differences in this activity between conditions or syllable positions, suggesting that differential carry-over is unlikely to account for the observed condition effects. However, the absence of systematic differences in carry-over across conditions does not establish that the baseline represents an uncontaminated pre-stimulus period. Thus, the resulting time-series estimates and pseudo-*F* values should be interpreted as responses relative to residual activity from the preceding auditory stimulus, and condition effects as differences between these baseline-referenced responses. Accordingly, the condition-specific effects should be interpreted with appropriate caution. Future work using longer interstimulus intervals or explicit deconvolution approaches could further clarify the contribution of overlapping neural responses.

A related consideration concerns the relatively short interstimulus interval used in the present design (see above). This choice was motivated by the statistical learning paradigm, in which temporally contiguous input is critical for supporting the formation of structured representations and promoting the perception of sequential input as cohesive units. While longer interstimulus intervals can reduce overlap between evoked responses, they may also weaken statistical learning effects by disrupting temporal continuity. We therefore adopted a design that balances these competing considerations. Future work could systematically vary inter-stimulus interval duration to further characterize its impact on neural responses and learning outcomes. Together, these considerations highlight both the strengths of MEG for capturing rapid neural dynamics in naturalistic auditory processing, and the methodological challenges inherent in studying fast temporal sequences during development.

In summary, although the role of social feedback in learning from infancy through adulthood has been established (Brown et al., 2016, Van Duijvenvoorde et al., 2008), the current study provides a novel perspective by demonstrating how social feedback, when incidental to a learning task, can significantly influence implicit learning processes in adolescents. An important open question is whether this sensitivity of implicit learning to social feedback is as robust across the lifespan or varies in interesting ways with development. Future research should address this question by investigating how faces affect learning at different developmental stages. Overall, the present findings indicate that feedback from human faces can alter broader cognitive processes, revealing a deep connection between social feedback, information processing, and human learning.

## CRediT authorship contribution statement

**Andrew N Meltzoff:** Writing – review & editing, Funding acquisition, Conceptualization. **Neva M Corrigan:** Writing – review & editing, Methodology, Data curation. **Samu Taulu:** Writing – review & editing, Methodology. **Julia C Mizrahi:** Writing – review & editing, Writing – original draft, Visualization, Project administration, Methodology, Investigation, Conceptualization. **Eric Larson:** Writing – review & editing, Visualization, Validation, Software, Methodology, Data curation. **Erica Peterson:** Writing – review & editing, Methodology, Investigation, Formal analysis, Data curation. **Katherine K Meltzoff:** Writing – review & editing, Methodology, Investigation. **Alexis N Bosseler:** Writing – review & editing, Writing – original draft, Visualization, Validation, Supervision, Project administration, Methodology, Investigation, Formal analysis, Data curation, Conceptualization. **Patricia K Kuhl:** Writing – review & editing, Funding acquisition, Conceptualization.

## Ethics declaration

Written informed consent to take part in the study and to publish the article has been obtained from all participants or their legal representatives. The privacy rights of participants have been observed.

This study was performed in compliance with relevant laws, regulatory frameworks and guidelines where the research took place. This study was approved by the University of Washington Institutional Review Board (IRB) / UW Human Subjects Division (HSD). (Approval No. STUDY00003236)

## Funding

This work was funded by a grant from the Bezos Family Foundation (PKK, ANM). The use of REDCap was supported by the Institute of Translational Health Sciences, which is funded by the National Center for Advancing Translational Sciences of the NIH under award number UL1TR002319.

## Declaration of Competing Interest

The authors declare that they have no known competing financial interests or personal relationships that could have influenced the work reported in this paper.

## Data Availability

I have shared a link to our data at the attach file step
